# Long-term culture of SH-SY5Y neuroblastoma cells in the absence of neurotrophins: A novel model of neuronal ageing

**DOI:** 10.1016/j.jneumeth.2021.109301

**Published:** 2021-10-01

**Authors:** Lisa Strother, Gareth B. Miles, Alison R. Holiday, Ying Cheng, Gayle H. Doherty

**Affiliations:** Bute Building, School of Psychology and Neuroscience, University of St Andrews, West Burn Lane, St Andrews, Fife KY16 9TS, UK

**Keywords:** **BDNF,**, Brain derived neurotrophic factor, **DAPI,**, 4’−6-diamidino-2-phenylindole, **DMEM,**, Dulbecco’s Modified Eagle Medium, **5-fdu,**, 5-fluorodeoxyuridine, **H**_**2**_**-DCFDA,**, 2',7'-Dichlorodihydrofluorescein diacetate, **HNE,**, 4-hydroxynonenol, **HRP,**, Horse radish peroxidase, **LDH,**, Lactate dehydrogenase, **MTT,**, 3-(4,5-dimethylthiazol-2-yl)− 2,5-diphenyltetrazolium bromide, **NeuN,**, Hexaribonucleotide Binding Protein-3, **NGF,**, Nerve growth factor, **PBS,**, Phosphate buffered saline, **ROS,**, Reactive oxygen species, SR-2**,**, Serum replacement – 2, Sv2**,**, Synaptic vesicle glycoprotein 2, TBS**,**, Tris buffered saline, TH**,**, Tyrosine hydroxylase, Ageing, Culture, Mitochondria, Neuroblastoma, Neuronal network, Oxidative stress

## Abstract

**Background:**

Studying human ageing is of increasing importance due to the worldwide ageing population. However, it faces the challenge of lengthy experiments to produce an ageing phenotype. Often, to recreate the hallmarks of ageing requires complex empirical conditions that can confound data interpretation. Indeed, many studies use whole organisms with relatively short life spans, which may have little, or limited, relevance to human ageing. There has been extensive use of cell lines to study ageing in human somatic cells, but the modelling of human neuronal ageing is somewhat more complex *in vitro*.

**New Method:**

We cultured the well-characterised SH-SY5Y human neural cell line to produce high purity cultures of cells differentiated to express a neuronal phenotype, and designed a protocol to maintain these cells in culture until they accumulated biomarkers of cellular ageing.

**Results:**

Our data validate a novel and simple technique for the efficient differentiation and long-term maintenance of SH-SY5Y cells, expressing markers of neuronal differentiation and demonstrating electrical activity in culture. Over time *in vitro*, these cells progressively accumulate markers of ageing such as enhanced production of reactive oxygen species and accumulation of oxidative damage.

**Comparison to Existing Methods:**

In comparison to existing techniques to model neuronal ageing our method is cost effective, requiring no specialist equipment or growth factors.

**Conclusions:**

We demonstrate that SH-SY5Y cells, grown under these culture conditions, represent a simple model of neuronal ageing that is amenable to cell biological, biochemical and electrophysiological investigation.

## Highlights

• Ageing study is often hindered by the need for complex and lengthy experiments.
• SH-SY5Y cells underwent neuronal differentiation and were cultured until they were of an aged phenotype.
• These cells were electrically active and acquired oxidative damage.
• This is a novel technique to model neuronal ageing *in vitro.*

## 1 Introduction

Human neuroblastoma cell lines, such as SH-SY5Y, are a widely used *in vitro* model system, and have been utilised for a plethora of neuroscience applications including cell viability assays (Jaworska-Feil et al., 2010, Funakohi-Tago et al., 2018), neuronal ultrastructure (Cheng et al., 2020), studies of neurophysiology (Tosetti et al., 1998; Öz and Çelik, 2016) and testing of pharmacological preparations (Cheung et al., 2009, Jiang et al., 2018). These cells were originally derived from the SK-N-SH cell line which was sub-cloned three times to SH-SY, SH-SY5 and finally to the SH-SY5Y cell line. SH-SY5Y cells are often used in their undifferentiated state and these cultures contain both floating and adherent cells. The adherent cells also adopt two distinctive phenotypes: neuroblast-like ‘N’ type cells, which express some neuron-specific biochemical markers such as dopamine-β-hydroxylase (Ross et al., 1983) and non-neuronal substrate adhesive cells, ‘S’ type cells which exhibit an epithelial-like phenotype and do not express any neuronal markers (Encinas et al., 2000). To overcome the issues of having a heterogeneous cell population, these cells can be induced, by a variety of protocols, to differentiate into a mature neuron-like phenotype. This is characterised by exit from the cell cycle, the formation of a rounded cell body coupled with extension of neurites and expression of neuron-specific markers such as βIII-tubulin, NeuN, neurofilament and synaptic vesicle marker Sv2 (Agholme et al., 2010). These cells also form synapses and demonstrate the ability to generate action potentials (Johansson, 1994, Öz and Çelik, 2016), and express both neurotransmitters (Lopes et al., 2010) and their receptors (Adem et al., 1987; Qian et al., 2018). Development of protocols to differentiate these cells continues with refinement of the commonly used retinoic acid protocol (Simões et al., 2021) and variations to the addition of trophic support (Jun et al., 2020) reported very recently.

Working with neural cell lines offers a number of advantages over primary neuronal culture. Deriving primary neurons from animals raises both cost and ethical issues, which is not the case with cell lines. In addition, a major advantage of working with SH-SY5Y cells is their human origin. Thus they express the human form of disease-relevant proteins within a cellular background of human protein and gene expression patterns. They can also be cultured in large quantities, which is advantageous for both biochemical manipulation and analysis.

However, many current protocols for culturing these cells in a differentiated state are either inefficient (resulting in undifferentiated cells being maintained within the culture; Filograna et al., 2015), involve the use of trophic factor support that can influence intracellular signalling cascades (Encinas et al., 2000) or are technically demanding (Constantinescu et al., 2007). Neuronal differentiation can be attained by a variety of methods including retinoic acid-evoked differentiation (Encinas et al., 2000; Simpson et al., 2001; Constantinescu et al., 2007; Cheung et al., 2009) or staurosporin (Prince and Oreland, 1997), alone or in combination with growth factors (Jämsä et al., 2004). For instance, retinoic acid (10 μM) induces growth inhibition of around 55% (compared to untreated controls) after 7 days of treatment suggesting that a significant number of cells remain in the cell cycle following retinoic acid exposure (Filograna et al., 2015). For fully differentiated cells, mitosis needs to be eliminated and thus retinoic acid alone is insufficient to fully differentiate these cells. To counteract this exogenous trophic factors such as BDNF are often used (Teppola et al., 2016).

Beyond differentiation, the lack of endogenous neurotrophic factors in neuronal cultures due to the absence of glial cells can result in apoptosis and therefore it has become common practice to add growth factors to culture media. However, the use of trophic factors is potentially problematic as signalling *via* these factors can protect cells from neurotoxicity and for the purposes of examining cell death and neuroprotection this can present a significant challenge. The neurotrophin, nerve growth factor (NGF), activates a PI3kinase/ Akt signalling cascade to promote cell survival (Yano and Chao, 2000) and thus determining the effects of exogenous neuroprotective agents on this signalling pathway is difficult if it is already activated. A diversity of potential therapeutics for neurodegeneration and spinal cord repair activate this signalling cascade including NGF *per se* (Fahnestock and Shekari, 2019), leptin (Doherty et al., 2008; Malekizadeh et al., 2017), and quercetin (Çiftçi et al., 2016, Zaplatic et al., 2019). The most commonly used neurotrophin in SH-SY5Y cultures is BDNF and indeed it is often used to help differentiate as well as support the cells in culture. However, inhibition of the cell cycle is unreliable when following this protocol (Teppola et al., 2016). Furthermore, like NGF, BDNF activates common survival pathways such as PI3kinase/ Akt (Segal, 2003).

A previous study detailed a method for culturing neuroblastoma cells without the need for trophic support (Constantinescu et al., 2007). Following retinoic acid differentiation, cells were exposed to a cocktail of mitotic inhibitors (fluorodeoxyuridine, cytosine arabinoside and uridine) and were incubated in a culture system wherein a perfusion pump was used to maintain the cells. However, although this generated pure differentiated cultures of SH-SY5Y cells, the perfusion pump introduces technical complexity. Furthermore, the use of the mitotic inhibitor cocktail introduces more confounding factors for cell viability. Cytosine arabinoside is neurotoxic to rat primary sympathetic neurons (Martin et al., 1990) and cerebellar granule cells (Paterson et al., 1998). In contrast, fluoroxyuridine has not been previously linked to neurotoxicity.

Here we present a simplified culture method, allowing differentiation and maintenance of SH-SY5Y neuroblastoma cells, using a combination of retinoic acid followed by 5-fluorodeoxyuridine to inhibit proliferation of undifferentiated cells. Maintaining the SH-SY5Y cells in defined culture medium allowed establishment of a neuronal phenotype and electrical signalling. Further, accumulation of oxidative damage markers, previously used to define an ageing phenotype for *in vitro* primary neurons (Lesuisse and Martin, 1992), occurred with long-term culture in these conditions. Thus we present a novel and simple, neurotrophin-free technique for efficient differentiation, and long-term culture, of SH-SY5Y cells and illustrate the development of an ageing phenotype in these cultures.

## 2 Materials and methods

### 2.1 Materials

Unless otherwise stated all chemical and reagents used in this study were purchased from Sigma Aldrich (UK).

### 2.2 Maintenance of SH-SY5Y cells

SH-SY5Y (European Collection of Cell Cultures) cells were defrosted and grown in accordance with cell bank protocols. Ethical permission to use these cells within the University of St Andrews for the purposes for medical and scientific research was obtained from the School of Psychology and Neuroscience ethics committee (Ref PS11189). Cells were grown to confluence in tissue culture flasks in Dulbecco’s Modified Eagle Medium (DMEM) supplemented with 10% iron-fortified new-born calf serum (Cosmic Calf Serum, Fisher, UK). Iron-fortified calf serum contains up to four times the amount of transferrin and available iron as fetal calf sera supporting the rapid proliferation of SH-SY5Y cells prior to differentiation as iron is crucial for the bioenergetics of proliferating cells (Pourcelot et al., 2015). Cells were trypsinised in 0.05% trypsin (Worthington’s, USA) in calcium and magnesium free buffer solution, and plated on either 96 well culture plates (10,000 cells per well; Nunc, vwr, UK) or on 0.0005% w/v poly-L-lysine-coated 13 mm diameter borosilicate glass coverslips (50,000 cells per coverslip), in DMEM supplemented with 4500 mg/L D-glucose, 10 mg/ml penicillin/ streptomycin and 10% iron-fortified new-born calf serum. All cells used in these experiments were of passage number 3–20.

### 2.3 Culture conditions

When cells had reached 70% confluence, they were stimulated with retinoic acid (10 μM) to induce differentiation to a neuronal phenotype, and the serum content of the medium reduced to 1%. After 5 days, medium was changed to one containing mitotic inhibitor (5-fluorodeoxyuridine (5-fdu; 18 μM (Malekizadeh et al., 2017)) and cells were maintained in this medium for the duration of the experiment, changing 100% of the medium every 3–4 days. When changing the medium, the cultures were washed twice in DMEM to remove any adherent dead cells. For experiments using serum-containing medium, this maintenance medium was DMEM supplemented with 4500 mg/L D-glucose, 10 mg/ml penicillin/ streptomycin, 18 μM 5-fdu and 1% iron-fortified new-born calf serum; and for experiments using defined medium, the cells were maintained in DMEM supplemented with 4500 mg/L D-glucose, 10 mg/ml penicillin/ streptomycin, 18 μM 5-fdu and 2% serum replacement −2 (SR-2; Sigma UK). Cells were kept in a humidified incubator at 37 °C and 5% CO_2_. Throughout this study, time in culture is expressed as number of weeks *in vitro* post removal of retinoic acid.

### 2.4 Immunocytochemistry

Cells were fixed in neutral buffered formalin for 15 min followed by repeat phosphate buffered saline (PBS) washes and blocking of non-specific antibody binding using PBS with 0.1% triton X-100 (PBS-T) and 10% heat inactivated horse serum. Cells were incubated in primary antibody at 4 ^o^C overnight at an appropriate dilution for each antibody (Table 1). Thereafter they were washed in PBS-T and incubated for one hour in the fluorescent secondary antibody at room temperature (Table 1). Coverslips were mounted in fluorescent mounting medium (1% w/v N-propyl-gallate dissolved 80% v/v glycerol; 20% v/v PBS) and imaged using a Zeiss Axio MR2 with Zen image analysis software.

**Table 1:** Details of antibodies used in these experiments. Details of dilutions, applications and company these antibodies were purchased from are given.

Antibody	Dilution	Application	Manufacturer
Mouse anti-βIII-tubulin	1: 1000	ICC	Promega, UK
Rabbit anti-4-hydroxynonenol	1:500	ICC	Insight Biotechnologies, UK
Mouse anti-synapsin	1:1000	ICC	Cell Signalling Technologies
Mouse anti-α-tubulin	1:5000	Western blot	Sigma Aldrich, UK

### 2.5 Electrophysiology

Cells were grown on poly-L-lysine-coated borosilicate glass coverslips in DMEM supplemented with 2% SR-2 for 4 weeks post differentiation. For electrophysiological recordings, coverslips containing cells were immersed in a recording chamber continuously superfused with artificial cerebrospinal fluid (approximately 1 ml per second; gassed with 95% O_2_ and 5% CO_2_ at room temperature, ~20 °C). Whole-cell patch-clamp recordings were established under infrared-differential interference contrast microscopy using borosilicated glass microelectrodes (3–5 MΩ) filled with intracellular solution. Signals were amplified and filtered (4 kHz low-pass Bessel filter) with a MultiClamp 700B amplifier (Molecular Devices, Sunnyvale, CA, USA) and acquired at ≥ 10 kHz using a Digidata 1440AA/D board and pClamp software (Molecular Devices). The recording aCSF contained (in mM): 127 NaCl, 3 KCl, 1.25 NaH_2_PO_4_, 1 MgCl_2_, 2 CaCl_2_, 26 NaHCO_3_, 10 glucose. The intracellular solution for patch-clamp recordings contained (in mM): 140 KMeSO_4_, 10 NaCl, 1 CaCl_2_, 10 HEPES, 1 EGTA, 3 Mg-ATP and 0.4 GTP-Na2 (pH 7.2–7.3, adjusted with KOH).

### 2.6 Determination of viability

Initial determination of cell wellbeing was carried out by visual inspection with phase contrast microscopy and recorded using a Nikon Coolpix digital camera linked to a Nikon TS100F inverted microscope. To estimate cell number per field of view, cells were fixed in neutral buffered formalin and labelled using 500 ng/ml 4’−6-diamidino-2-phenylindole (DAPI) in PBS before imaging under a Zeiss Axio MR2 fluorescent microscope. Data from 5 randomly selected fields of view from 7 independent experiments and 2 durations of culture were collected and presented as average number of DAPI positive nuclei per field of view. Lactate dehydrogenase (LDH) is a cytoplasmic enzyme that is released upon cell membrane rupture and thus the concentration of LDH in the culture medium can be used to determine the level of cell death in a culture. Determination of LDH levels in the culture medium was carried out as described previously (Oldreive et al., 2010).

### 2.7 Determination of mitochondrial redox capacity

To ascertain mitochondrial redox capacity, mitochondrial activity was analysed *via* the 3-(4,5-dimethylthiazol-2-yl)−2,5-diphenyltetrazolium bromide (MTT) assay as described previously (Doherty, 2007). The resulting purple azo-dye was detected at 570 nm with a Biohit BP800 plate reader. Data was expressed as percentage mitochondrial activity relative to that detected in cells 2 weeks post differentiation.

### 2.8 Western blot

Proteins from cultures were extracted into 500 µl tris-buffered saline containing protease inhibitor cocktail (Set II, Merck, UK). Protein samples were run on a 10% SDS-polyacrylamide gel (NuSep) giving a final protein concentration per well of 20 µg/µl as determined by the Bradford assay, and transferred to a nitrocellulose membrane. Membranes were blocked in 5% w/v dried skimmed milk in Tris buffered saline (TBS) + 0.1% v/v triton X-100 prior to incubation with the mouse-anti-alpha tubulin antibody overnight at 4 °C. Primary antibody binding was detected using a horseradish peroxidase (HRP)-conjugated secondary antibody at 1:10,000 v/v in TBS-T and visualised using chemiluminescence ECL substrate (Pierce) and imaged with a GelDoc Imager it2 with Vision Works software.

### 2.9 Oxyblot

Protein samples were chemically denatured by the addition of 12% w/v sodium dodecyl sulphate in deionized water for 15 min. The samples were then mixed with dinitrophenyl residues according to the manufacturer’s protocol (Merck, UK)**,** and incubated for 20 min, then neutralisation buffer was then added to halt the reaction. 10 µg/µl of each sample was loaded onto a gel and the remainder of the assay was carried out as per the manufacturer’s instructions with the intensity of the bands determined using Image J software.

### 2.10 Statistical analysis

Results are presented as mean ± standard error of the mean of triplicate samples from at least three separate experiments. Data was screened for normality using a Kolmogorov–Smirnov test. Statistical significance was determined using students *t*-test where data was normally distributed and Wilcoxon signed ranked test for non-parametric data.

## 3 Results

### 3.1 Differentiated SH-SY5Y cells treated with mitotic inhibitor are viable in culture for 4 weeks

**Fig. 1:**
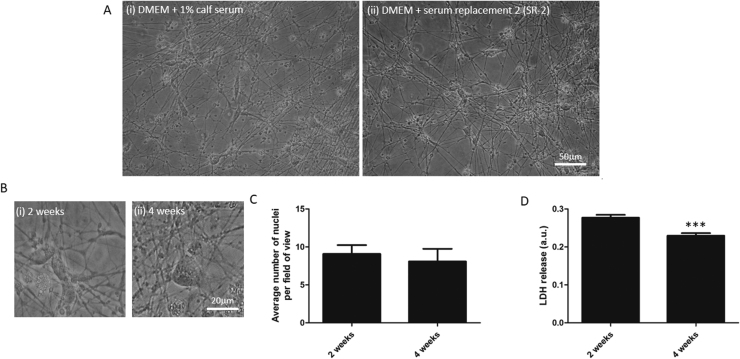
Differentiated SH-SY5Y cells treated with mitotic inhibitor are viable in culture for 4 weeks. (A) Phase contrast photomicrographs of SH-SY5Y cells 2 weeks post differentiation with retinoic acid and maintained in 1% iron-fortified calf serum-containing (Ai) or SR-2-supplemented medium (Aii). Phase bright cell bodies and extensive networks of processes are observed revealing viable cells with a neuronal morphology. Images are taken at 40x magnification and scale bar represents 50 µm. (B) Images of neurons maintained in SR-2-supplemented medium for 2 weeks (Bi) or 4 weeks (Bii) post differentiation. (C) Bar chart of DAPI counts of cell nuclei in culture 2 week and 4 week post-differentiation and (D) bar chart of lactate dehydrogenase release into the culture medium at these time-points. The means and standard errors of the means of seven experiments established in triplicate are shown. *** represents statistical significance (P < 0.001).

To determine whether differentiated SH-SY5Y cells could survive in long term culture without exogenous neurotrophic support, and in the absence of a peristaltic pump, cultures of retinoic acid-treated cells were followed for up to 4 weeks post-differentiation. These cells were maintained in either SR-2-supplemented, 1% iron fortified calf serum-supplemented or serum-free medium with mitotic inhibitor. Two weeks after retinoic acid withdrawal there were clear differences in the number of cells surviving in the cultures (Fig. 1A) with no cells surviving in serum-free cultures (not shown; n = 3). However, 2 weeks post-differentiation there were viable cells with a neuronal morphology and robust process outgrowth in cultures maintained in either 1% iron-fortified calf serum (Fig. 1Ai) or SR-2-supplemented cultures (Fig. 1Aii). After 4 weeks in culture, the on-going survival in cultures maintained in 1% serum was unreliable with 60% of cultures retaining viable neurons at 4 weeks post differentiation, and 40% of cultures in which all neurons had died prior to this (n = 5). In contrast, cells maintained in DMEM supplemented with 2% SR-2 remained viable 4 weeks after withdrawal of retinoic acid. Under phase contrast microscopy, phase bright cell bodies and multiple processes are observed in SR-2-maintained cultures imaged both 2 weeks (Fig. 1Bi) and 4 weeks (Fig. 1Bii) after retinoic acid treatment ended. There was also a marked increase in the number of processes visible in the cultures between 2 and 4 weeks. To further examine the cell number at these two time-points, we stained cultures with the DNA dye, DAPI and counted the number of DAPI positive nuclei per field of view (63x magnification) in SR-2-supplemented cultures. Analysis of these counts revealed that there was no significant difference in the number of cells per field of view between 2 and 4 weeks (9.086 + 1.157 at 2 weeks and 8.068 + 1.676 at 4 weeks; n = 7 plate downs; *P* > 0.05; Fig. 1C). To further investigate levels of cell death within these cultures, LDH release into the culture medium, as a measure of membrane integrity, was determined. There were significantly less dying cells in the 4 week cultures (0.23 ± 0.007 a.u.) compared to 2 week cultures (0.28 ± 0.008 a.u.; n = 7; P < 0.001; Fig. 1D). Thus levels of cell death are not increasing with time *in vitro* implying that the cell population has stabilised by this time in culture. We conclude that retinoic acid-differentiated SH-SY5Y cells are viable for at least 4 weeks after cessation of retinoic acid treatment when cultured in DMEM supplemented with 2% SR-2 and 18 μM 5-fdu. This is the medium that was used for all further experiments.

### 3.2 Differentiated SH-SY5Y cells treated with mitotic inhibitor express neuron specific markers

**Fig. 2:**
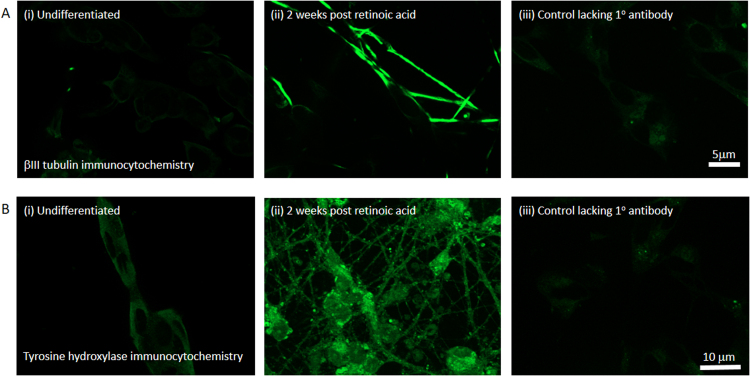
Differentiated SH-SY5Y cells treated with mitotic inhibitor express neuron specific markers. (A) Images of undifferentiated (Ai) and cultures 2 weeks post-differentiation (Aii) immunocytochemically labelled to detect βIII-tubulin expression, and negative control image (Aiii). Images were taken at 63 x magnification and scale bar represents 5 µm. (B) Images of undifferentiated (Bi) and 2 week cultures (Bii) immunocytochemically labelled to determine tyrosine hydroxylase expression, and negative control image (Biii). Images were taken at 40x magnification and scale bar represents 10 µm.

Although it is well known that exposure to retinoic acid induces differentiation and the expression of neuron-specific markers in SH-SY5Y cells (Constantinescu et al., 2007, Korecka et al., 2013, Simões et al., 2021), the expression of these markers in our system remained to be determined; this information gives an indication of the purity of the cultures. Therefore SH-SY5Y cells were fixed for immunocytochemistry when undifferentiated, or 2 weeks after retinoic acid-induced differentiation and the expression of βIII-tubulin detected using immunocytochemistry. Clear immunofluorescence can be seen in βIII-tubulin-stained cultures in contrast to the control condition in which the primary antibody has been omitted. Analysis of images stained with βIII-tubulin revealed that virtually all of the differentiated cells in our cultures expressed this neuronal marker whereas staining levels in the undifferentiated cells were similar to those seen in the control lacking primary antibody (Fig. 2A). In addition, because retinoic acid differentiation is known to induce a dopaminergic phenotype (Korecka et al., 2013), we stained the cultures for the presence of tyrosine hydroxylase (TH), the rate-limiting enzyme in dopamine biosynthesis. Robust TH immunoreactivity was detected in the differentiated cultures at 2 weeks (Fig. 2Bii) whereas the levels in the undifferentiated cells (Fig. 2Bi) were much less. Thus our protocol induces the differentiation of these cells to produce neuronal markers and the expression of these is maintained once retinoic acid is withdrawn.

### 3.3 Cells in these cultures form synapses and exhibit electrophysiological activity

**Fig. 3:**
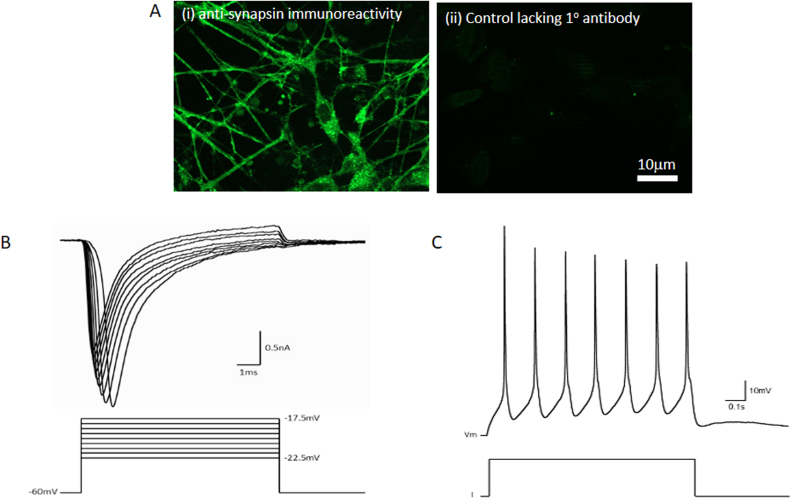
Cells in these cultures form synapses and exhibit electrophysiological activity. (A) Images of cultures 2 weeks post-differentiation (Ai) immunocytochemically labelled to determine synapsin expression and negative control image (Aii). Images were taken at 40 x magnification and scale bar represents 10 µm. (B&C) Representative whole-cell patch-clamp recordings from cells 2 weeks post differentiation showing (B) inward sodium currents in response to depolarising voltage steps and (C) repetitive firing of action potentials in response to current injection. Voltage-clamp and current-clamp protocols are depicted beneath raw data in (B) and (C).

One of the defining features of a neuronal population is the ability to form synaptic connections and a functional neuronal network. Immunocytochemical staining for the synaptic marker, synapsin, was used to determine whether these cells formed synapses with one another after 2 weeks in culture. Clear punctate immunolabelling was observed on the processes extended by the fully differentiated SH-SY5Y cells (Fig. 3Ai) in contrast to the negative control lacking primary antibody (Fig. 3Aii). Undifferentiated SH-SY5Y cells do not show this pattern of immunoreactivity (Supplementary Fig. 1). This indicates that by 2 weeks post retinoic acid exposure the cells are expressing a synaptic marker in a pattern that suggests the formation of functional synapses.

Whilst the presence of synaptic markers indicates that the SH-SY5Y cells are electrically active under these culture conditions, it does not provide evidence of neurons with the functional capacity to assemble neuronal networks. Therefore we next performed whole-cell patch-clamp electrophysiological recordings from cultures of 4 week old cells to assess their functional properties (Fig. 3B–C). Recordings performed in voltage-clamp mode demonstrated fast-inactivating sodium currents in response to a series of depolarising voltage steps (10 ms duration) performed from a holding potential of −60 mV (Fig. 3B). Recordings conducted in current-clamp mode revealed that differentiated SH-SY5Y cells are able to generate repetitive trains of action potentials in response to depolarising current steps (1 s duration; Fig. 3C). Taken together, these data demonstrate that these cells have differentiated into a functional neuronal phenotype.

### 3.4 SH-SY5Y cells exhibit decreased mitochondrial activity and increased levels of ROS with time in culture

**Fig. 4:**
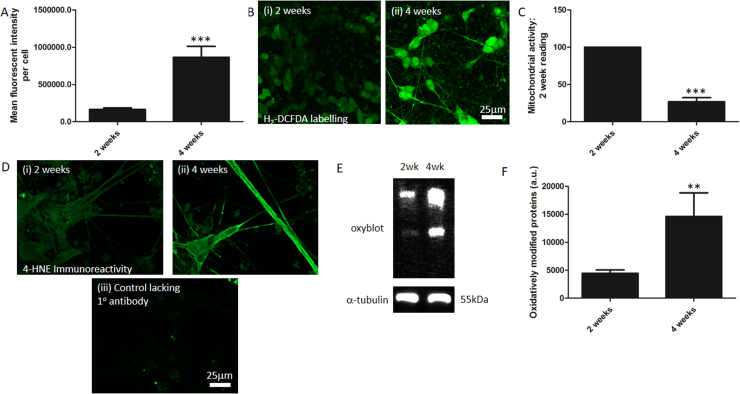
SH-SY5Y cells exhibit decreased mitochondrial activity and increased levels of ROS with age. (A) Quantification of fluorescent intensity in H_2_-DCMFA labelled cultures 2 weeks and 4 weeks post-differentiation. The means and standard errors of the means of 8 separate experiments established in duplicate and with at least five fields of view imaged per coverslip are shown. *** represents statistical significance (P < 0.001). (B) Images of 2 (Bi) and 4 (Bii) week cultures stained with the fluorescent dye H_2_-DCFDA to demonstrate ROS levels. Images were taken at 20 x magnification and scale bar represents 25 µm. (C) Bar chart of relative levels of mitochondrial redox capacity in cultures 2 weeks and 4 weeks post-differentiation. The means and standard errors of the means of 6 separate experiments are shown. *** represents statistical significance (P < 0.001). (D) Images of cultures immunocytochemically labelled to determine HNE expression cultures 2 weeks (Di) and 4 weeks (Dii) post-differentiation and negative control image (Diii). Images were taken at 40 x magnification and scale bar represents 25 µm. (E) Representative image of an oxyblot to detect protein oxidation in cultures 2 weeks and 4 weeks post-differentiation and image of a corresponding western blot of these samples demonstrating α-tubulin expression as a loading control. (F) Quantification of the amount of oxidatively modified protein in cultures 2 weeks and 4 post-differentiation. The means and standard errors of the means of 11 separate experiments are shown. ** represents statistical significance (P < 0.01).

A number of methods have been proposed to age primary neurons in culture (Lesuiss and Martin, 2002; Burrinha et al., 2021). Although there is no definitive time point at which cultures can be considered aged, the progressive accumulation of oxidative damage with time is proposed as a valid *in vitro* model of the *in vivo* build-up of oxidatively damaged cellular constituents observed in ageing ( Kruman et al., 1997; Valavanidis et al., 2009). To determine if there was an increase in ROS generation over time in our cultures, we used the fluorescent dye H_2_-DCFDA (10 µM), which fluoresces when oxidised by ROS. Cells were imaged at 2 and 4 weeks post retinoic acid differentiation using confocal microscopy (Fig. 4B), and the mean fluorescent intensity per cell measured at each time-point (Fig. 4A). The results show a clear increase in the intensity of H_2_-DCFDA fluorescence between 2 and 4 weeks post differentiation detecting 862,500 ± 149,700 a.u. per cell in 4 week cultures. This is significantly higher than that observed in 2 week cultures (164,100 ± 21,170; n = 8 separate cultures; P < 0.001) Thus levels of ROS generation increase with time in culture.

Mitochondria are the major site of ROS generation within the cell, and with age, mitochondrial redox capacity is reduced, which goes some way to explain why ROS levels rise with age and damage cellular constituents (Liang and Godley, 2003). This can be measured using MTT assay (Shearman et al., 1995). Thus we ascertained mitochondrial redox function in the cells 2 and 4 weeks post differentiation. The data reveal that there was a 73.25 + 5.62% decrease in mitochondrial redox capacity between 2 and 4 weeks *in vitro* (Fig. 4A; n = 7; P < 0.001), which cannot be attributed to a decrease in neuronal viability (see Fig. 1). These cultures exhibit a decrease in redox capacity between 2 and 4 weeks post differentiation coupled to a concomitant rise in ROS generation.

### 3.5 SH-SY5Y cells exhibit accumulation of oxidative damage with time in culture

Although reduced redox capacity and enhanced generation of ROS have been linked to ageing (Liang and Godley, 2003), it is the accumulation of oxidatively-modified intracellular components that is believed to underpin the phenotypic alterations associated with age (Vanhooren et al., 2015).

To determine whether we observe enhanced lipid peroxidation with time in culture, immunocytochemistry was used to detect 4-hydroxynonenol (HNE). Formation of HNE results from oxidation of phospholipids containing ω-6 polyunsaturated fatty acyl chains and therefore HNE presence is a widely used as a marker of oxidatively damaged lipids (Spickett, 2013). Samples were stained 2 or 4 weeks after retinoic acid differentiation and a clear increase in immunoreactivity between the two time points was observed (Fig. 4Di-ii), and enhanced immunoreactivity is seen at both timepoints compared to controls lacking the primary antibody (Fig. 4D).

Protein oxidation is a well-established feature of ageing and can be detected using oxyblot to identify carbonyl groups introduced into proteins by oxidative reactions mediated by ROS. There is a marked increase in the intensity of protein bands derived from 4 week as compared to 2 week cultures (Fig. 4E). Quantification of the band intensity from oxyblot revealed that there was a significant increase in protein oxidation between the 2 time-points. Thus a lane intensity of 4438.3 + 595.8 a.u. was detected for 2 week protein samples and of 14598.10445 + 4219.4 for 4 week protein samples representing around a 3-fold increase in protein oxidation (n = 11; P < 0.01). Taken together our data demonstrate that these cells are accumulating oxidative damage over time in culture.

## 4 Discussion

The neuroblastoma cell line SH-SY5Y, is commonly used in neurodegeneration research as they express a neuronal phenotype and, as a human cell line, avoid concerns regarding cross-species effects, solely expressing human proteins (Cheung et al., 2009, Zhang et al., 2010). Retinoic acid-induced differentiation is by far the best categorised protocol for differentiation and promotes upregulation of neuronal markers and neurite outgrowth (Jämsä et al., 2004, Korecka et al., 2013, Simões et al., 2021). By using retinoic acid as a differentiating agent and a simple protocol thereafter to eliminate any remaining undifferentiated cells, we have established a highly pure culture of cells expressing a neuronal phenotype with the functional capacity to form a neuronal network, and which can be maintained *in vitro* for extended periods until they accumulated age-related biomarkers.

Previous research has suggested that whilst differentiating the cells with retinoic acid gives rise to a neuronal phenotype, not all cells exit the cell cycle (Filograna et al., 2015). However, subsequent treatment with growth factors gives rise to reduction in mitosis, promotes neurite outgrowth and increased expression of neuron specific markers such as MAP2, NeuN and βIII-tubulin (Encinas et al., 2000, Constantinescu et al., 2007, Agholme et al., 2010). Furthermore, addition of growth factors increases synaptic vesicle marker expression which is indicative that these cells are capable of firing action potentials. Therefore, in isolation retinoic acid is useful to induce differentiation but additional culture conditions are required for long term maintenance.

For undifferentiated cells, fetal bovine serum is used to supplement culture media as it encourages cell proliferation and contains a cocktail of growth factors which support survival and proliferation (Gstraunthaler, 2003). Here we use iron-fortified calf serum due its higher levels of free iron and transferrin further supporting the cells during the proliferative phase (Pourcelot et al., 2015). To encourage the differentiation of cell lines, serum concentration is routinely reduced to between 1% and 5% inhibiting proliferation and supporting differentiation (Ferruzza et al., 2013). In this study we lowered the iron-fortified calf serum concentration from 10% to 1% during the differentiation phase of the protocol and thereafter switched cells into medium that was either serum-free, contained 1% iron-fortified calf serum or a commercially available serum replacement (SR-2), which has previously been shown to support primary neurons *in vitro* (Doherty et al., 2013). We have shown that optimal cell survival was achieved using this defined serum replacement and there is no need for non-defined serum-containing media or for supplementation with exogenous growth factors. This also reduces the use of animals in the generation of reagents for such studies. It has been widely reported that without subsequent treatment with neurotrophins such as NGF or brain-derived neurotrophic factor (BDNF) these cultures exhibit enhanced levels of cell death and lower expression of neuron specific markers (Jämsä et al., 2004, Agholme et al., 2010). In contrast we do not observe a marked increase in cell death between 2 and 4 weeks in culture suggesting that within this timeframe these cells are able to survive in the absence of neurotrophic support. Finally, the use of growth factors in cell culture, especially for long term culture, is a costly endeavour and therefore we opted to develop a protocol without exogenous trophic factor support. To conclude, we have shown that even in the absence of neurotrophin supplementation we have given rise to fully differentiated neural culture which expresses neuronal and synaptic markers and that remain viable across 4 weeks in culture.

Importantly, to verify that our cells were excitable, a defining feature of neurons, we conducted electrophysiological recordings. We found that our cells express appropriate ion channels and are capable of firing repetitive trains of action potentials in response to current stimuli. This reveals that this culture model is useful for generating highly pure neuronal cultures that are amenable to sensitive functional analyses, which have been used to reveal some of the earliest perturbations in models of progressive neurodegenerative diseases such as Amyotrophic Lateral Sclerosis (e.g. Devlin et al., 2015).

Current theories of ageing suggest that as neurons age, they accumulate damage through the activities of ROS and thus express higher levels of oxidatively-modified DNA, RNA and proteins, rendering them less efficient or inactive. A variety of models are currently used to study ageing *in vivo* including longitudinal human studies such as the Framingham cohort (Elias et al., 2000), rodent (Liao and Kennedy, 2014), avian (Travin and Feniouk, 2016) and invertebrates (Torgovnick et al., 2013) and all of these require extended experimental protocols due to complexity and/or lifespan. The use of cell culture models of ageing in non-neuronal cells is widespread (Salama et al., 2014, Childs et al., 2015) but the use of ageing primary neuronal cultures has not been widely adopted by the scientific community. Long term maintenance of neuronal cultures can be problematic and involve complex culture media, which can confound data analysis from these models. Furthermore, the need to use animal donors of this tissue for these experiments adds the additional factors of ethical issues and the nature of cells, in that lack human-specific proteins. Using the method developed herein, it is now possible to study the underlying mechanisms of cellular ageing and to examine potential pharmacological means of preventing the deleterious effects of ageing in human cells. Thus, we have demonstrated that these cells show signs ageing such as oxidative damage to both protein and lipids as well as a decrease in mitochondrial redox capacity and an increase in the generation of ROS.

As neurons are post-mitotic cells, the potential for neurons to show senescence has received little attention in the scientific literature as in their post-mitotic state they cannot exhibit replicative senescence. Whilst our cells are also post-mitotic and we similarly did not address senescence here, this model could be used to investigate neuronal senescent markers in future studies. For instance, post-mitotic mature neurons can develop a senescence-like phenotype. Thus mature neurons of aged mice exhibit DNA damage, activated p38MAPkinase, high ROS production and oxidative damage, interleukin IL-6 production, heterochromatinization and senescence-associated β-galactosidase activity, with the number of these senescence-like neurons increasing with age (Jurk et al., 2012). With evidence that both Alzheimer’s-associated tau (Musi et al., 2018) and amyloid (Wei et al., 2016) can induce cellular senescence, investigations of their effects on these aged neurons, with respect to senescent markers could prove interesting.

An emerging model for studying neurons *in vitro* is the use of inducible pluripotent stem cells (iPSCs), which can be differentiated to a neuronal phenotype. These can have additional benefits over the SH-SY5Y cells as they can be derived from both patients and healthy volunteers (for neurological disorders). This technology has the potential to accurately model human disease *in vitro*, however these cultures require extensive, expensive and complex protocols. Furthermore as this technology remains under development the transformation efficiency for a given cell type can be as low as 1% (Robinton and Daley, 2012). Thus current iPSC cultures are heterogeneous to varying degrees whether containing a mix of neurons and glia or of neuronal sub-types (Srikanth and Young-Pearse, 2014). Furthermore, the reprogramming required to establish iPSC cultures re-sets the cells to an embryonic phenotype which raises issues for modelling late-onset disorders. Attempts have been made to overcome this, for instance expressing progerin in these cells to induce phenotypic ageing in Parkinson’s models (Miller et al., 2013). Despite telomeric shortening being most commonly associated with aging of somatic cells, downregulation of telomerase and telomere shortening during neural differentiation allows iPSCs to be converted to neurons that more accurately model the aged neurons most commonly affected by neurodegeneration (Vera et al., 2016). Finally, to circumvent the need to re-programme to the embryonic state, the direct transcription factor-based conversion of fibroblasts into induced neurons (iNs) is emerging as a promising means of establishing aged human neurons *in vitro* (Pang et al., 2011) and these cells retain transcriptomic profiles akin to their aged origins (Mertens et al., 2015). Together these findings suggest that iPSC technology still offers a valuable avenue in modelling human neurological disorders *in vitro* and better techniques for working with these cells are emerging regularly.

Another interesting and emerging technology of importance to neuroscience is the development of three-dimensional (3D) culture systems, which are usually based on iPSCs. These are complex systems wherein post-iPSC differentiation organoids form (which can be facilitated by exogenous scaffolds (Mansour et al., 2018)) and these are 3D self-organized structures with both morphological and functional characteristics that are similar to the brain (Papaspyropoulos et al., 2020). Whilst the technology continues to develop no specific attempts to age these organoids have been reported and the complexity of the system makes these time-consuming and technically demanding to develop.

In contrast our SH-SY5Y cultures can be rapidly and inexpensively cultured in large numbers and obtain phenotypic markers of ageing within a few short weeks whilst requiring minimal maintenance and manipulation. We propose that they offer a simple and inexpensive model for biochemical and cellular investigations of ageing *in vitro*.

## Research Data Statement

The data presented in this article is available at: https://doi.org/10.17630/f901900e-bebc-472d-930d-01956ac508c6.

## CRediT authorship contribution statement

**Lisa Strother:** Methodology, Formal Analysis, Investigation, Writing – original draft. **Gareth B Miles:** Validation, Formal analysis, Investigation, Writing – review & editing. **Alison R. Holiday:** Validation, Writing – review & editing. **Ying Cheng:** Validation, Writing – review & editing. **Gayle H. Doherty:** Conceptualization, Writing – review & editing, Visualization, Supervision, Project administration.
