# Supplementary figures and images for: Long-term culture of SH-SY5Y neuroblastoma cells in the absence of neurotrophins: A novel model of neuronal ageing
Source: J Neurosci Methods. 2021 Oct 1;362:109301. doi: 10.1016/j.jneumeth.2021.109301 (PMC8434422; doi:10.1016/j.jneumeth.2021.109301)

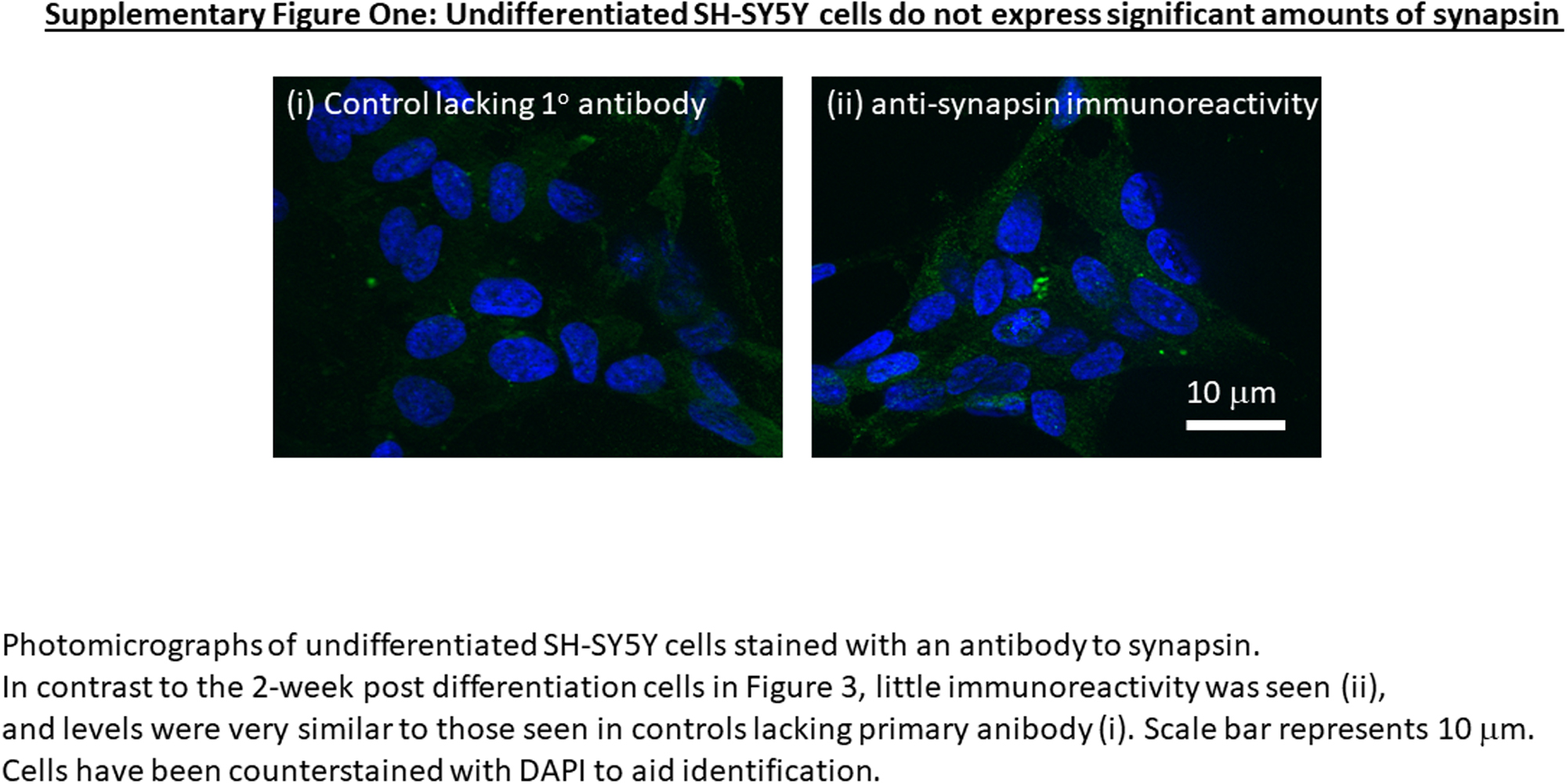

Supplement: Supplementary file 1 — Supplementary material [file mmc1.jpg]
